# Applying binary mixed model to predict knee osteoarthritis pain

**DOI:** 10.1371/journal.pone.0325678

**Published:** 2025-07-15

**Authors:** Helal El-Zaatari, Liubov Arbeeva, Amanda E. Nelson

**Affiliations:** Thurston Arthitis Research Center, University of North Carolina School of Medicine, Chapel Hill, North Carolina, United States of America; University College London, UNITED KINGDOM OF GREAT BRITAIN AND NORTHERN IRELAND

## Abstract

Data used to understand knee osteoarthritis (KOA) often involves knee-level, rather than person-level information. Failure to account for the correlation between joints within a person may lead to inaccurate inferences. The aim of this study was to develop a flexible, data-driven framework for predicting knee pain outcomes, incorporating the advantages of both random forest (RF) and mixed effects models for correlated data. Specifically, we utilized data from the baseline visit of the Osteoarthritis Initiative (OAI) and applied the Binary Mixed Models (BiMM) algorithm to predict two binary dependent variables. 1) presence of knee pain, stiffness or aching in the past 12 months and 2) presence of knee pain indicated by a KOOS pain score > 85. This novel approach was compared to standard random forests (RF), which do not account for correlations among knees. This study demonstrates the potential of BiMM as a predictive tool for KOA pain, achieving a comparable or slightly improved performance over traditional RF models while simultaneously accounting for within-person correlation among knees. This is a significant advancement, as most machine learning models to date have only considered each knee individually. These findings support the integration of BiMM in KOA outcome prediction, providing a nuanced alternative to existing models and advancing our understanding of important KOA outcomes on the person level. Although demonstrated here for KOA, this method is relevant to any situation where within-person correlations are relevant, including other joints and other musculoskeletal conditions.

## Introduction

Knee osteoarthritis (KOA) is a prevalent chronic disease that affects over 21% of American adults, significantly impacting quality of life and functional mobility [1]. As the disease progresses, patients often experience persistent and debilitating pain, which is a primary driver of reduced physical activity, psychological distress, and healthcare utilization [2]. Effective pain management and prediction are crucial for improving patient outcomes and guiding clinical decision-making.

KOA pain prediction models have the potential to serve as valuable tools in this context. By providing clinicians with actionable insights, these models can aid in tailoring treatment strategies to individual patients, prioritizing interventions for those at higher risk of severe pain, and optimizing resource allocation [3]. Furthermore, as early screening tools, pain prediction models can identify individuals who may benefit from preventive measures, such as weight management, physical therapy, or pharmacological interventions, before the disease significantly worsens [4].

KOA-related patient outcomes may differ among individuals with unilateral and bilateral KOA [5]. Pain and social impacts from bilateral KOA worsen with increasing body burden, making pain management more complex . Clinical trials are often designed to study a homogeneous sample in which only one knee is affected by OA [6]. Many cohort-based studies also do not take into account whether the pain is unilateral or bilateral [7]. However, radiographic and self-reported knee-specific measures can be impacted by contra-lateral knee, which should be taken into account in the relevant analyses [8].

Despite their advantages, most existing knee OA pain prediction models fail to address a critical aspect of the disease: the inherent correlation between the two knees of an individual. Since OA often affects both knees, either symmetrically or asymmetrically, the pain and disease progression in one knee are frequently influenced by the condition of the other. Ignoring this correlation can lead to biased predictions and suboptimal model performance, as most Machine Learning (ML) methods assume the knees are independent units of observation. This limitation underscores the need for innovative approaches that explicitly account for within-person knee correlation, improving the accuracy and reliability of pain predictions in knee OA.

The earliest pain prediction models were based on conventional logistic regression methods, treating each knee as an independent unit of observation [9]. Following the landmark work of Zhang *et al*., the importance of accounting for correlation between an individual’s knees became evident, leading to the adoption of methods such as Generalized Estimating Equations (GEE) and Polychotomous Logistic Regression. By incorporating this correlation, significant improvements were achieved in both prediction performance and the interpretability of variables [9]. This distinction between individual-level and knee-level effects provided novel insights into the interplay of these factors in knee osteoarthritis.

Currently, pain prediction models fall into two primary categories. The first category comprises traditional regression models, which heavily rely on logistic regression, sometimes enhanced with Bayesian or LASSO techniques [10]. In some cases, these logistic regression-based models account for correlation by including random effects, effectively functioning as GEE models. However, the review by Ramazanian *et al*. leaves it unclear whether all such models consistently accounted for correlation.

The second category consists of machine learning models employing classification techniques such as Artificial Neural Networks, K-Nearest Neighbors, Support Vector Machines, and Random Forests (RF) [10]. Their potential in terms of predictive performance is enormous due to the ability to process multi-dimensional data and to account for complex interactions among different features. However, the lack of reproducibility of results published in earlier papers and limited interpretability of results have led to skepticism about ML approaches in clinical research. Often, the researchers argue that conventional statistics focuses on accurate and reliable parameter estimates [11]. One of the strongest arguments, particularly in KOA research, is that most ML methods are inherently unable to account for correlation among the dependent variables. This limitation poses challenges in modeling clustered data, such as bilateral knee observations, where accounting for correlation is critical.

This highlights the need for a method capable of delivering accurate pain prediction while properly accounting for within-person knee correlation. Several RF-based methods have been developed to handle clustered dependent variables [12]. These methods accommodate continuous, discrete, and binary clustered dependent variables. Among them, those designed for continuous and discrete variables primarily target longitudinal data, whereas the Binary Mixed Effects Model (BiMM) specifically addresses clustered binary dependent variables, and is most relevant for the use case described in this paper. Its tailored approach makes it well-suited for the unique data structure present in this research context, ensuring both accurate prediction and proper handling of within-person knee correlation. This represents a novel application of a ML method integrated with a traditional regression model for KOA pain prediction, uniquely designed to account for within-person knee correlation.

## Methods

### Data source and preparation

The data for this study were extracted from the Osteoarthritis Initiative (OAI), a ten-year multicenter observational study of men and women sponsored by the National Institutes of Health. The dataset is publicly available, anonymized, and can be accessed under a data use agreement at https://nda.nih.gov/oai. Data from the OAI baseline visit were used and processed as described by Nelson *et al*. (2022) [13].

To prepare the dataset, redundant variables and those collected from a small subset of individuals were removed. Unordered categorical variables were transformed into binary indicators for each unique category, while ordered categorical variables were encoded as integers. Continuous variables were left untransformed and no attempts were made to normalize them to a standard normal distribution. Rows with missing values were excluded and the analysis included only individuals with data available for both knees. Since the primary outcome was the presence of knee pain, stiffness, or aching in the past 12 months, variables directly related to knee pain, such as the Knee Injury and Osteoarthitis Outcomes Score (KOOS) pain score and the Western Ontario and McMaster Universities Arthitis Index (WOMAC) pain score, were excluded to avoid overlap with the outcome. The final dataset consisted of 92 variables, encompassing 5718 knees from 2859 individuals. Of these, 89 variables were used as independent predictors in the random forests within BiMM and standalone random forests. The ID and knee side variables were included as random effects in the mixed effects model, leaving the binary outcome variable as the 92nd variable.

### Outcomes

Two binary pain outcomes were used in this study. The first is the presence of knee pain, stiffness, or aching in the past 12 months. The second is KOOS pain score categorized such that a score above 85 is considered as no knee pain [14].

### Binary mixed model

Both a RF and a BiMM were trained and tested on this dataset, which was split into a 70/30 ratio for training and testing at the individual level. The BiMM alternates between fitting a random forest with all independent variables and using the resulting probabilities to inform a mixed-effects model that accounts for clustered binary outcomes. The process begins with the BiMM fitting a standard random forest on the binary dependent variable using the training dataset [15]. Once the RF is trained, the fitted classification probabilities are obtained.

To address the correlation between knees, a Bayesian logistic mixed-effects model is then fitted. In this model, the random forest output probabilities serve as fixed effects with uninformative priors. The model incorporated a random intercept for each individual as a random effect, addressing the correlation found for each knee within an individual, with covariance structured using Normal and Wishart prior distributions. The model is expressed as:

$$logit(Y_{it})=\beta_{0}+\beta_{1}RF(Xit)+Z_{it}b_{it}$$ (1)

Here *Y*_*it*_ represents the binary dependent variable of presence of knee pain,stiffness or aching in the past 12 months. *i* represents the individual with and *t* represents the side of the knee (*t* = 1 for the left side and *t* = 2 for the right side). The term $\beta_{0}$ is the intercept, while $RF(X_{it})$ denotes the random forest probabilities for individual *i* and side *t*, derived from 89 independent variables $X_{it}$. The fixed effect is $\beta_{1}$ and *Z*_*it*_ represents interaction between ID and side with random effect *b*_*it*_.

Using the model in Equation (1), we extract predicted probabilities, *Q*_*it*_, for individual *i* and knee side *t*. These probabilities are then used to update the binary dependent variable by combining the extracted probability *Q*_*it*_ with the original dependent variable *Y*_*it*_. his update is performed using a split function, h(·), such that the updated variable takes the form $Y_{it}^{*}=h(Y_{it}\;+\;Q_{it})$. With the updated *Y*_*it*_ a standard random forest and Bayesian mixed effect model is trained again. This procedure is repeated iteratively, with each iteration refining the model, until the change in the posterior log likelihood from the Bayesian mixed-effects model falls below a specified tolerance threshold.

### Optimal split function

There are three split functions provided by Speiser *et al*. [15]. The first split function is defined below $h_{1}(Y_{it}\;+\;Q_{it})=\left\{ \begin{array}{cc} 0 & Y_{it}+Q_{it}\leq k_{1}\\ 1 & Y_{it}+Q_{it}>k_{1} \end{array}\right.\;\;\;$. The output of this function depends on the value of the constant *k*_1_. This constant acts as a threshold and can take on values from 0 to 1. The split function can turn dependent variable values of 0 into a 1 but not the other way around. This provides a mechanism for maximizing the sensitivity of the predictions. The second split function is given by $h_{2}(Y_{it}\;+\;Q_{it})=\left\{ \begin{array}{cc} 0 & Y_{it}+Q_{it}<k_{2}\\ 1 & Y_{it}+Q_{it}\geq k_{2} \end{array}\right.\;\;\;$. The third split function is a combination of both *h*_1_ and *h*_2_ with threshold constants *k*_1_ = 0.5 and *k*_2_ = 1.5. It is given by $h_{3}(Y_{it}\;+\;Q_{it})=\left\{ \begin{array}{cc} 0 & Y_{it}+Q_{it}<0.5\\ 1 & Y_{it}+Q_{it}>1.5\\ 1 & \text{with probability}\;Q_{it}\;\text{if}\;0.5<Y_{it}+Q_{it}<1.5\\ 0 & \text{with probability}\;1-Q_{it}\;\text{if}\;0.5<Y_{it}+Q_{it}<1.5 \end{array}\right.\;\;\;\;\;$. Values of 0 can turn into a 1 and values of 1 can turn into a 0 therefor this split function does not maximize sensitivity or specificity.

To identify the optimal split function and its corresponding threshold constant, we first fixed the training and test sets. For the first split function, *h*_1_, we varied the threshold constant, *k*_1_, from 0.05 to 0.95 in increments of 0.05, recording the resulting AUC at each step. Similarly, for the second split function, *h*_2_, we adjusted the threshold constant, *k*_2_, from 1.05 to 1.95 using the same step size, tracking the area under the curve (AUC) values. The split function and its corresponding threshold constant that yielded the highest AUC were selected. This process was conducted separately for each of the two binary dependent variables.

The Random Forest (RF) and BiMM models were trained and tested using 100 distinct data partitions to evaluate predictive performance and variable importance. Each partition produced a unique training and test set, resulting in 100 pairs. For the BiMM, the optimal split function and threshold constant were selected.

For the binary dependent variable representing the presence of knee pain, stiffness, or aching in the past 12 months, a class weight of 0.37 was applied to both the standalone RF and the RF within the BiMM to address class imbalance. This weighting gave the minority class (presence of knee pain) 2.7 times more influence during the training process. For the binary KOOS pain score variable, both classes were assigned equal weights. The optimal split function for the knee pain variable was *h*_1_ with a threshold constant of 0.25, while the KOOS pain score variable used *h*_2_ with a threshold constant of 1.5. Note that varying the threshold constant for the split function yielded negligible change in area under the receiver operating characteristic curve (AUC) values.

Predictive performance was evaluated using the AUC. Variable importance was assessed by identifying the top 20 most influential variables for each model based on their Mean Decrease Gini scores. Both AUC values and the top 20 influential variables were recorded for each data partition. The average AUC was calculated for RF and BiMM to facilitate a comparison of their predictive performance. A two-sample t-test was performed to determine if the difference in mean AUC values between the two models was statistically significant.

To compare variable importance, the average Mean Decrease Gini score across the 100 partitions was calculated for the top 10 variables in each model. Additionally, the total number of unique influential variables identified across the 100 partitions was counted for both models. It was not possible to use SHAP values to assess variable importance in the final random forest model within the BiMM framework. This limitation arises because BiMM updates and modifies the binary dependent variables during each iteration, reducing the informativeness of SHAP values, which rely on the specific values of the dependent variable. In contrast, the Mean Decrease Gini measure remains robust to these updates, as it is a node-based metric rather than one that depends directly on the outcome values.

## Results

### Sample characteristics

Overall there were 2859 patients in this data set. The sample was typical of KOA studies, with a mean age of 61 years, BMI in the overweight range, and a majority were women and White (Table 1).

**Table 1:** Characteristics of included individuals from the OAI baseline cohort (n = 2859).

Characteristics	Mean ± SD or n (%)
Age, years (mean ± SD)	61.2 ± 9.1
BMI, kg/*m*^2^ (mean ± SD)	28.4 ± 4.6
Women	1630 (57.0%)
White	2459 (86.0% )
Annual Income > $50, 000	1822 (63.73%)
Currently employed	1804 (63.10%)

This study examined two dependent variables, with their correlation assessed using the phi coefficient. The phi coefficient for the frequency of knee pain, stiffness, or aching over the past 12 months was 0.3, while the phi coefficient for the binary KOOS pain score was 0.31, indicating a weak relationship between the two sides [16]. A total of 89 independent variables were included in the analysis. For a comprehensive list and details on the types of independent variables used, refer to Nelson *et al*. (2022) [13].

### Presence of knee pain, stiffness or aching in the past 12 months

For the binary outcome of knee pain, aching, or stiffness in the past 12 months, BiMM had a numerically better predictive accuracy than Random Forest (RF) by 1%, achieving an AUC of 0.7415 compared to RF’s 0.7313. The 95% confidence interval for the difference, obtained via a two sample t-test, was given by [0.0070,0.01334].

**Fig 1:**
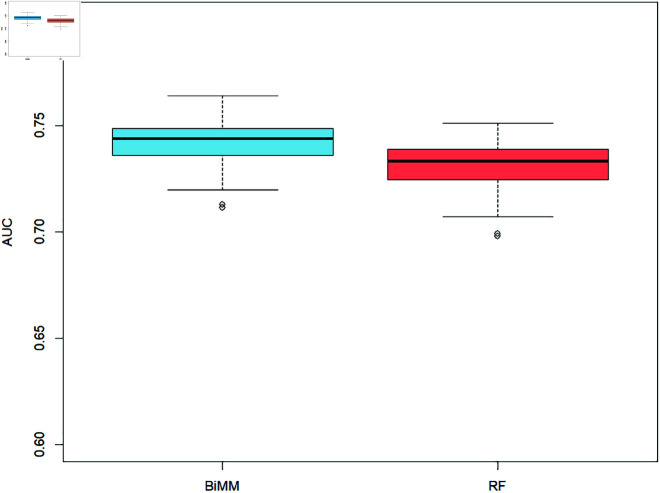
AUC values for Bimm and RF methods across the 100 data partitions for presence of knee pain, aching or stiffness in the past 12 months.

The difference in predictive performance is visually apparent in the box plot (Fig 1) as the median AUC line of the BiMM lies outside the AUC inter-quartile range of the RF, however it is not clinically meaningful. The range of AUC values across the 100 partitions for the BiMM were between 0.7113 and 0.7640. For the random forest, the range of AUC values across the 100 partitions were between 0.6978 and 0.7510. The standard deviation for the AUC values of the BiMM was 0.0110 and the standard deviation for the RF was 0.0116.

We report the mean accuracy, sensitivity, and specificity for both BiMM and RF across 100 iterations. For the knee pain binary outcome, we used a probability threshold of 0.78, where values below 0.78 indicate no knee pain, and values above 0.78 indicate knee pain. This threshold was chosen to keep the false positive rate below 30%. As shown in the table below, the BiMM model required an average of 10 to 11 iterations to converge with an error tolerance of 0.5. The mean sensitivity for BiMM was 0.635, substantially higher than the 0.415 sensitivity of RF, a difference of over 20 percentage points. However, this improvement in sensitivity came at the cost of specificity; BiMM achieved a mean specificity of 0.719, whereas RF had a higher specificity of 0.882. This difference is also reflected in the false positive rates, with BiMM exhibiting a rate of 0.28—nearly double that of RF (0.117). Despite this, BiMM achieved better overall accuracy (0.658) compared to RF (0.546). In summary, RF performed better at correctly identifying no knee pain cases but struggled with predicting knee pain, while BiMM was more effective at detecting knee pain cases but had a higher rate of false positives. The performance metrics discussed above can be found in Table 2.

**Table 2:** Performance metrics for BiMM vs. RF with presence of knee pain, stiffness or aching in the past 12 months dependent variable.

Method	Mean AUC	Mean Iterations	Mean Sensitivity	Mean Specificity	Mean Accuracy	Mean False Positive Rate
BiMM	0.743 ± 0.011	10.50 ± 5.03	0.635 ± 0.020	0.719 ± 0.018	0.658 ± 0.011	0.280 ± 0.018
RF	0.713 ± 0.012	NA	0.415 ± 0.020	0.882 ± 0.013	0.546 ± 0.014	0.117 ± 0.013

**Fig 2:**
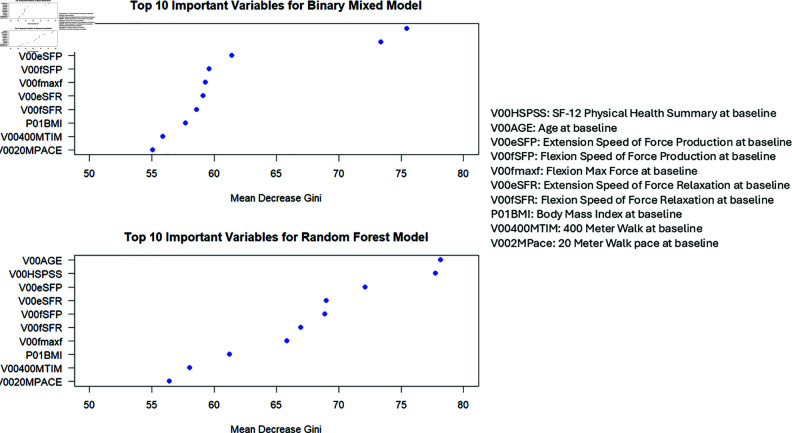
Average mean decrease Gini across 100 partitions for the top 10 most important variables for binary mixed model and random forest.

Over 100 partitions, BiMM identified 23 unique variables within the top 20 lists, compared to 21 unique variables selected by RF. Interestingly, the Mean Decrease Gini scores for the top 10 variables were consistently lower in the BiMM model than in RF (Fig 2).

This indicates that RF, by failing to account for within-person knee correlations, may overestimate variable importance, potentially compromising prediction accuracy in cases where such correlations are relevant. BiMM’s superior performance underscores its ability to effectively incorporate knee-level correlations, enhancing predictive accuracy for this outcome.

### Binary KOOS pain score

The mean AUC for BiMM was 0.8243 and the mean AUC for RF was 0.8244 which represented a negligible difference of 0.0001. This difference was not statistically significant as determined by a two sample t-test. The two sample t-test resulted in a t-statistic value of -0.025567 with an associated p-value of 0.9796. The 95% confidence interval for the difference was given by [−0.002732,0.002662]. The range of AUC values across the 100 partitions for the BiMM were between 0.8018 and 0.8470. For the random forest, the range of AUC values across the 100 partitions were between 0.8013 and 0.8463. The standard deviation for the AUC values of the BiMM was 0.009764 and the standard deviation for the RF was 0.00957. For the KOOS binary pain outcome we applied a threshold of 0.5 in order to maintain a false positive rate below 30%. The results for BiMM and RF on the KOOS outcome were more similar, with comparable AUC, sensitivity, specificity, accuracy, and false positive rates. Additionally, unlike the other knee pain outcome, the false positive rates and sensitivity values were closely aligned for both models. This suggests that, for the KOOS outcome, neither model had a clear advantage in predicting knee pain or no knee pain cases, unlike the previous dependent variable where BiMM and RF exhibited distinct strengths and weaknesses. Another notable difference for this KOOS outcome was that BiMM required only three iterations to converge at an error tolerance of 0.5, compared to the 10 to 11 iterations needed for the other knee pain outcome. The performance metrics discussed above can be found in Table 3.

**Table 3:** Performance metrics for binary KOOS pain score dependent variable.

Method	Mean AUC	Mean Iterations	Mean Sensitivity	Mean Specificity	Mean Accuracy	Mean False Positive Rate
BiMM	0.824 ± 0.010	3 ± 0	0.592 ± 0.019	0.871 ± 0.011	0.775 ± 0.009	0.128 ± 0.012
RF	0.824 ± 0.010	NA	0.542 ± 0.020	0.901 ± 0.009	0.778 ± 0.009	0.098 ± 0.009

Over 100 partitions, BiMM identified 23 unique variables within the top 20 lists, compared to 24 unique variables selected by RF. The Mean Decrease Gini scores for the top 10 variables were similar in both the BiMM model and RF model.

## Discussion

One of the primary limitations in ML studies of KOA, compared to conventional regression models and tests, is the lack of adjustment for the correlation between knees within an individual. This limitation is frequently highlighted by reviewers but has yet to be widely addressed by the research community [17]. During our review of the literature on machine learning methods applied to KOA, we found that it was rare for studies to acknowledge, let alone address, the fact that knees within the same person are correlated.

The only consistent approach to this issue appeared in studies using generalized estimating equations (GEE), which explicitly model correlation within knees. To bridge this gap, there is the need to develop and adopt the methods that address this limitation and raise awareness within the OA research community. Zhang *et al*. first extended conventional regression models to handle correlated data in seminal GEE paper [9], which was applied to OA outcomes and significantly improved the statistical rigor of OA research [9]. Although there are many published methods for handling clustered data within the ML approach[12], these are all relatively recent and likely to be unfamiliar to the OA community. Our paper demonstrates an example of such an approach within a specific category of machine learning models: random forests.

Moreover, for other machine learning techniques commonly used in KOA research, such as support vector machines and neural networks, there are methods or configurations available to account for correlated dependent variables [18, 19]. By showcasing these methods, we aim to encourage the broader adoption of techniques that properly account for correlations, thereby improving the rigor and reliability of KOA research.

The BiMM is applicable to any correlated binary dependent variable, making it highly adaptable to scenarios within and beyond the KOA context. For example, MRI features are often graded in different sub-regions within the same knee. This method can address correlation within subregions within the same joint, multiple joints within one person. While we have showcased its utility in KOA, the adaptability of BiMM extends to other musculoskeletal conditions involving multiple correlated joints. This versatility highlights the potential for broader applications and impactful findings across various fields of study.

Overall there were negligible differences in predictive performance and variable importance for the BiMM and RF across both binary dependent variables. The BiMM method demonstrates performance levels nearly identical to those of the Random Forest (RF), with the added benefit of accounting for correlation within knees. As the degree of correlation in clustered binary data increases, BiMM is more likely to outperform RF in prediction accuracy. Recall, the correlation for both knee pain outcomes were weak, which might explain why the performance levels of BiMM and RF were nearly identical. If the correlation between the clustered binary variables had been stronger, it is plausible that BiMM would have outperformed RF by better accounting for this correlation. This expectation is supported by a study by Speiser *et al*. (2019) that demonstrated that BiMM significantly outperformed standard RF in datasets with strong within-subject correlation under linear and tree-based data-generating structures [15]. These findings support the notion that BiMM offers a more appropriate modeling strategy than RF in highly clustered settings.

The overall weak correlation observed between knees in this dataset (phi coefficients of 0.3 and 0.31) likely limited the performance difference between the two methods. Future research should explore the potential of BiMM in datasets with stronger correlations and investigate enhancements to its split function to better capture the complex relationships between dependent variables. This study represents a novel application of machine learning integrated with traditional regression modeling, paving the way for more sophisticated and accurate predictive models in knee osteoarthritis research.

The advantage of BiMM over RF lies in its split function, which iteratively updates predictions by incorporating estimated random effects at each iteration [15]. This split function acts as an indicator function that integrates the estimated random effects into its domain, enabling BiMM to account for the correlation between knees effectively. By modifying the domain of the split function to better capture this correlation, predictive performance in BiMM could be further improved. Exploring such modifications remains an area for future research.

Additionally, we did not investigate causal data-generating mechanisms, as this was not the focus of the current study. While RF provides a flexible framework for prediction and classification, a standardized approach to minimizing bias and accounting for data complexity (such as clustering or nesting) has yet to be established and requires further research [20].

## Conclusion

This study provides a straightforward method of incorporating within-person knee correlation in pain prediction models for knee osteoarthritis. While traditional Random Forest (RF) models provide robust predictions, they fail to address the correlation between knees within individuals, a critical characteristic of knee osteoarthritis data. When this correlation is of particular interest or importance, the Binary Mixed Model (BiMM), with its iterative integration of random effects, offers a promising or confirmatory alternative, especially when the degree of correlation in the data is substantial.

## Supporting information

OAI Data. (CSV)
